# Machine Learning Support for Decision-Making in Kidney Transplantation: Step-by-step Development of a Technological Solution

**DOI:** 10.2196/34554

**Published:** 2022-06-14

**Authors:** François-Xavier Paquette, Amir Ghassemi, Olga Bukhtiyarova, Moustapha Cisse, Natanael Gagnon, Alexia Della Vecchia, Hobivola A Rabearivelo, Youssef Loudiyi

**Affiliations:** 1 BI Expertise Quebec, QC Canada; 2 Research Institute McGill University Heath Centre Montreal, QC Canada

**Keywords:** machine learning, artificial intelligence, medical decision support, kidney transplantation

## Abstract

**Background:**

Kidney transplantation is the preferred treatment option for patients with end-stage renal disease. To maximize patient and graft survival, the allocation of donor organs to potential recipients requires careful consideration.

**Objective:**

This study aimed to develop an innovative technological solution to enable better prediction of kidney transplant survival for each potential donor-recipient pair.

**Methods:**

We used deidentified data on past organ donors, recipients, and transplant outcomes in the United States from the Scientific Registry of Transplant Recipients. To predict transplant outcomes for potential donor-recipient pairs, we used several survival analysis models, including regression analysis (Cox proportional hazards), random survival forests, and several artificial neural networks (DeepSurv, DeepHit, and recurrent neural network [RNN]). We evaluated the performance of each model in terms of its ability to predict the probability of graft survival after kidney transplantation from deceased donors. Three metrics were used: the C-index, integrated Brier score, and integrated calibration index, along with calibration plots.

**Results:**

On the basis of the C-index metrics, the neural network–based models (DeepSurv, DeepHit, and RNN) had better discriminative ability than the Cox model and random survival forest model (0.650, 0.661, and 0.659 vs 0.646 and 0.644, respectively). The proposed RNN model offered a compromise between the good discriminative ability and calibration and was implemented in a technological solution of technology readiness level 4.

**Conclusions:**

Our technological solution based on the RNN model can effectively predict kidney transplant survival and provide support for medical professionals and candidate recipients in determining the most optimal donor-recipient pair.

## Introduction

### Current State of Organ Allocation

Deceased organ donation is the most common type of kidney donation [1] and can be defined as donation after neurological death (neurological determination of death [NDD]) and donation after circulatory death (DCD) [2]. Despite being authorized in Canada since 2006, DCD donations represented only 17% of deceased organ donations in Canada in 2012 [3]. The number of patients waiting for organ transplantation greatly exceeds the number of organs donated [4]. Ensuring an optimal donor identification and referral process and improving efficiency in identifying compatible donors would help avoid missed donation opportunities [3] and increase the rate of DCD [4]. Assisting informed decision-making regarding the acceptance of donor kidney by helping patients to better understand the treatment options and potential transplant outcomes would promote better treatment efficiency [5].

In current clinical practice, several kidney allocation algorithms are used to match donor organs with potential recipients. In the United States, the Organ Procurement and Transplantation Network uses a list of potential recipients that are ranked according to objective medical criteria (eg, blood type, tissue type, and size of the organ as well as medical urgency, time spent on the waiting list, and distance between the donor and recipient) [6]. Several simple numerical tools have also been implemented to guide kidney allocation. An example is the Estimated Post Transplant Survival score [7]. This score is assigned to all adult candidates on the kidney transplant waiting list and is based on 4 factors: candidate’s time on dialysis, current diagnosis of diabetes, prior solid organ transplants, and candidate’s age. The kidney donor risk index [8] combines various donor factors to summarize the risk of graft failure after kidney transplantation into a single number. It uses features such as donor’s age, height, weight, ethnicity (or race), history of hypertension, history of diabetes, cause of death, serum creatinine level, hepatitis C status, and DCD criteria. The kidney donor risk index is then remapped to a percentile scale where the lower percentiles (0%-20%) represent a lower risk of graft failure. Candidates with Estimated Post Transplant Survival scores ≤20% will receive offers for kidneys from donors with Kidney Donor Profile Index scores ≤20% before other candidates at the local, regional, and national levels of distribution [9]. Similar candidate and donor variables have also been considered in Canadian kidney allocation systems [10]. According to the recommendations of the Canadian Council for Donation and Transplantation, priority should be given to young recipients (especially when the organ donor is also young), donor-recipient pairs with zero mismatch for HLA ABDR, highly sensitized patients, and those requiring combined transplants.

### Machine Learning Support for Organ Donation

When deciding the suitability of a kidney graft for a recipient, it is important to estimate how long the donated organ will remain functional. To address this question, numerous studies have used machine learning (ML) models to predict kidney transplant outcomes, each differing in variable and outcome definitions.

Some models were built using data from either living donor [11] or deceased donor transplants only [12,13], whereas others considered both donor types [14].

In 2010, Reinaldo et al [15] evaluated several simple and interpretable ML models, in which the decision tree model showed 94% accuracy in predicting graft survival 1 year after transplant.

A recent study by Luck et al [16] proposed a neural network model built on data from the Scientific Registry of Transplant Recipients (SRTR) database, where the outcome of interest was graft failure. A total of 436 different variables were used to build the neural network model. The survival predictions were evaluated using a C-index (the percentage of transplant pairs correctly ordered by the model according to the observed survival durations), which was slightly higher than that obtained using the Cox model (0.655 compared with 0.65).

These studies built and evaluated various ML models; however, their termination at the stage of proof of concept makes it difficult to use the results for assistance in clinical decision-making.

Several tools have reached advanced technological readiness levels. Patzer et al [14] built a mobile app to predict 1- and 3-year patient survival using multivariate logistic regression analysis. Kilambi et al [17] quantified the benefits of accepting a kidney transplant based in part on the expected patient survival using Cox regression models. Loupy et al [18] designed a tool to predict long-term kidney allograft failure to guide posttransplant care, also using a Cox model. To the best of our knowledge, all published results are based on linear models that may not capture the nonlinear relationships between the input variables.

The *objective* of this project is to develop an innovative solution of technology readiness level 4 (TRL-4; component and validation in a laboratory environment) that would use ML to support medical decisions about accepting kidney transplants for particular donor-recipient pairs, with specific attention to DCD donations.

This study describes all stages of development of the ML technological solution: data acquisition and preparation, training and evaluation of ML models, and deployment of the solution.

## Methods

### Data Access and Data Security

BI Expertise obtained permission from SRTR (United States) to access its extensive historical data on organ transplants that were previously used in research [1,19].

Special measures were taken to maintain both the confidentiality and security of personal data. The BI Expertise team leveraged Microsoft Azure public cloud to ensure that all the data were secured and only the team could access it remotely. Data exfiltration risk was avoided by disabling all direct remote accesses. The environment was only visible to end users using a virtual machine inside Azure. This virtual machine was entirely isolated from the computers that were accessing it (no cut and paste).

The predictive modeling environment was based on the Azure ML data science platform and all the data resided in Azure Synapse Analytics. Both platforms were fully integrated to optimize the data preparation process and feature engineering activities. Once the predictive model was built and validated, it was deployed to a specific virtual machine that also hosted the user interface, which was accessible through a browser using a computer, tablet, or mobile device.

#### Ethics Approval

The proposed architecture was approved by the SRTR research ethics board (REB 2020-020H), and upon deployment, BI Expertise agreed to submit it to unannounced audits.

### Data Set

This study was based on several data tables from SRTR, namely, *DONOR_DECEASED*, *REC_HISTO*, *CAND_KIPA*, *TXF_KI*, and *TX_KI*. The tables contained individual deidentified sociodemographic and medical characteristics of kidney donors and recipients as well as outcomes of kidney transplantation *such as graft failure, recipient death, or loss to* follow-up.

We included first-time kidney recipients who underwent transplantation between January 1, 2000, and December 31, 2019. This choice of subset was motivated by important progress made in the field of kidney transplantation at the beginning of the year 2000, and the chosen data set included transplants after these changes were made. In addition, by selecting recipients from the same transplant era, we ensured that all recipients would have undergone similar methods of matching donor-recipient pairs [20].

### Data Cleaning and Selection of Variables

The selection of variables to be used for survival analysis was based on expert knowledge, data completeness, and previously published studies [12,21,22]. The input variables included sociodemographic characteristics of donors and recipients, history of comorbidities, blood type, details on donors’ death and levels of creatinine, time on the waiting list for recipients, and number of HLA mismatches. These data are typically known before the decision-making about the transplant and therefore can be reliably used as input for the ML mode. The exclusion criteria were the following: (1) variables not known before the transplantation (ie, immunosuppression therapy), (2) variables specific for the US medical system (ie, payment source for transplant recipients), and (3) variables with >20% of missing observations. Multimedia Appendix 1 provides a complete list of the variables and their definitions.

### Outcome Definition

The primary outcome was death-censored kidney graft survival, defined as the time elapsed between transplantation and diagnosis of graft failure. Data were censored at the time of the most recent follow-up for recipients who still had functioning grafts, at the time of their last record for those who were lost to follow-up, and at the time of death for those who died before experiencing graft failure. Probability of graft survival was predicted at set time points ranging from 0 to 15 years after transplantation, with intervals of 3 months between each time point*.*

### Feature Engineering

Some variables contained duplicate information, such as racial and ethnic groups. In this case, they were regrouped into a single variable. This resulted in the creation of new variables, which are described in detail in Multimedia Appendix 1.

LassoCV, ElasticNetCV, and recursive feature elimination feature selection methods from the scikit-learn package were used to select the most important variables.

### Survival Analysis Models

Several linear and nonlinear survival models were considered.

#### Cox Proportional Hazards

The Cox proportional hazards model [23] evaluates the effects of covariates on survival time and is commonly used in multivariate survival analysis because of its ease of implementation and interpretation. The Python package *scikit-survival* was used in this study to perform computations related to the Cox model.

#### DeepSurv

DeepSurv is a variant of the Cox model [24] that handles nonlinear data. The hazard ratio is produced by a neural network, which enables the model to learn from the interactions between covariates. The Python package *pycox* was used to perform training and testing of the DeepSurv model.

#### DeepHit

DeepHit [25] is an artificial neural network whose output vector is the joint probability distribution of all possible events (graft failure in this study) at each time point, which enables the model to learn the time-varying effects of each covariate on graft survival. The Python package *pycox* was used to perform training and testing of the DeepHit model.

#### Random Survival Forest

Random survival forest (RSF) [26] is an extension of the random forest model [27] that takes into account right-censoring of survival data. An RSF is an ensemble of survival trees, and each tree is grown on a subsample of the training data. The Python package *scikit-survival* was used to build and test the RSF model.

#### Recurrent Neural Network

##### Overview

The structure of our recurrent neural network (RNN) was inspired by previous studies that described deep recurrent survival analysis [28] and RNN-SURV [29]. The RNN presented in this study was implemented in Python using *TensorFlow 2.2* (Google Inc).

##### Structure of the RNN Model

**Figure 1:**
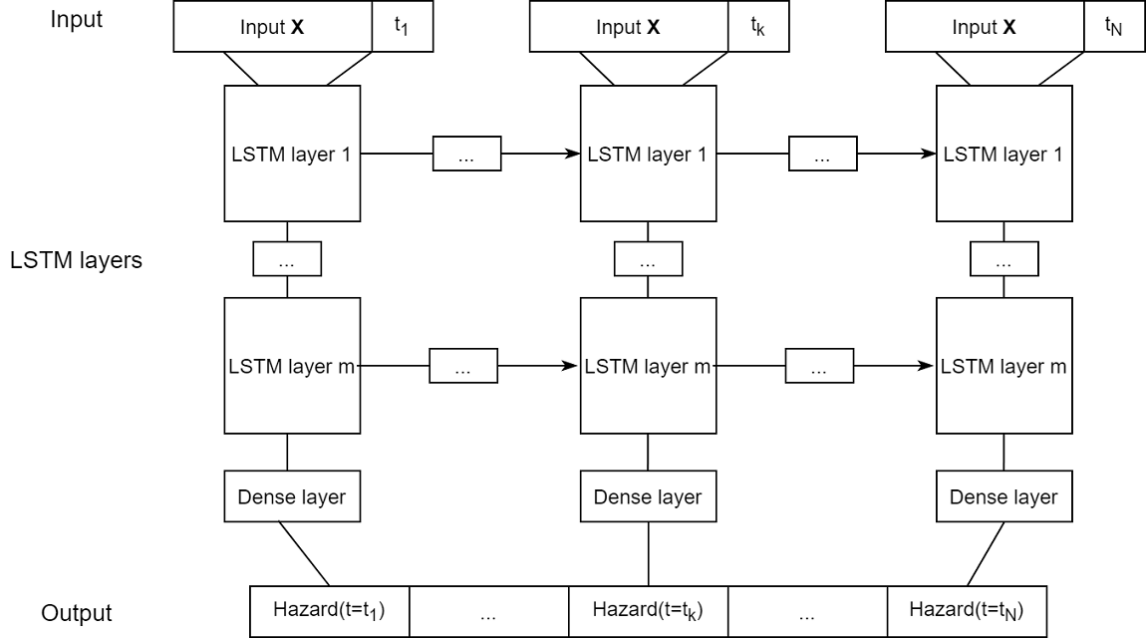
Structure of the recurrent neural network model. LSTM: long short-term memory.

For each of the N time intervals, the covariate vector X is passed, along with the time interval value *t*, through a series of m long short-term memory layers (Figure 1). The time interval value is added to explicitly capture the time-varying effects of the covariates. The N outputs are then passed through a dense layer with sigmoid activation to obtain the hazard rate at each time step. The hazard rates can be used to compute the estimated probability of survival at any time step *t* as follows:



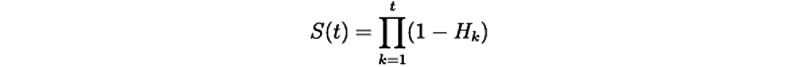



##### Loss Function

We compared 2 variants of loss functions, namely, the negative log-likelihood of the cumulative distribution function on all samples added to the negative log-likelihood of the probability density distribution on uncensored samples [28] and the ranking loss proposed in DeepHit [25].

##### Postprocessing of the RNN Output

To increase the calibration (refer to the *Model Performance* *Evaluation Metrics* section), a method to use the outputs of the RNN (individual hazard rates) as relative risk factors was devised, similar to the individual risk scores obtained from a Cox model. The main difference is that the risk factors vary over time. Therefore, for each patient, we interpreted the hazard rates at each time step as a risk score. From these risk scores, we aimed to obtain calibrated hazard rates to produce better calibrated survival predictions.

One approach to predict the hazard rates from the Cox model risk scores is as follows:



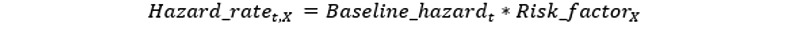



Where the baseline hazard can be estimated from the training data with:



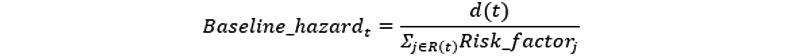



Where *d(t)* is the number of events at *t* and *R(t)* is the risk set at *t*, composed of all individuals still susceptible to the event of interest at time *t* [30].

A similar method was implemented for our RNN model, with the modification that the risk scores at each time step are associated with one of n risk bins, with each risk bin having its own baseline hazard. The cutoff points for the risk bins are determined by computing the n-quantiles of the estimated risk scores of the training samples at each time step.

Calibrated_Hazardi,t = Ri,t * BHk,t: estimated calibrated hazard rate for transplant i at time step t

*R_i,t_*: risk score for individual *i* at time step *t*

*BH_k,t_*: baseline hazard for risk bin *k* at time step *t*

Where the baseline hazards are estimated from the training data with:



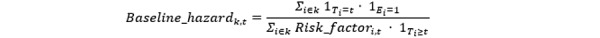



which represents the number of observed events at time *t* for samples of bin *k*, divided by the sum of risk scores at time *t* for samples of bin *k* that are still susceptible to the event of interest at time *t*.

The individual calibrated hazard rates can then be used to compute survival probabilities.

### Training and Evaluation Data Sets

The results presented in this study were obtained using 5-fold cross-validation. It consists of randomly splitting the data set into 5 partitions of equal size and repeating the training and evaluation process 5 times, each time using one partition (20%) as the evaluation set and the remaining (80%) as the training set.

Training and evaluating for hyperparameter tuning, choice of loss function, and choice of training approach were performed using 5-fold cross-validation (each with different permutations of the 5-fold partitions). These steps were performed on the same set used to compare ML models.

### Model Performance Evaluation Metrics

#### Concordance Index

The concordance index [31] is a measure of the discrimination power of a model. It measures the concordance between the ranking of the predicted risk metrics (eg, risk score, failure time, or probability of failure) and the observed failure times for all pairs of transplants. A pair of samples *i,j* is concordant if the predicted risk score of *i* is greater than that of *j* and sample *i* has a shorter survival period than *j*. The C-index is the number of concordant pairs of transplants divided by the total number of comparable pairs. The result can take any value between 0 and 1, with 0.5 representing no discrimination (random predictions) and 1 representing a perfect model.


Harell C-index = 
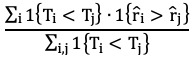
 where 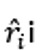 is the risk score for transplant *i*.


As the C-index uses a single time-independent risk metric to rank the transplants, it fails to account for the time-dependent effects of covariates on the risk of a patient. In the case of proportional hazard models such as Cox, this has no incidence (ie, risk scores do not change over time). However, for models that output individual survival distributions, the estimated risk of patients may vary with time. For example, a patient with a higher failure probability than others at an earlier time point might have a lower failure probability than others later on. Therefore, the time-dependent concordance index was used to evaluate the models [32]. For this index, a pair of transplants *i,j* is considered concordant if *i* experienced failure at a time *t_i_* sooner than *t_j_* and the probability of *i* surviving beyond *t_i_* is lower than that of *j* surviving beyond *t_i_*.


Antolini time-dependent C-index = 
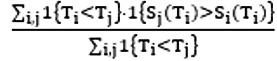



#### Integrated Brier Score

The Brier score [33] for a time point *t* is the average squared distance between the predicted probability of surviving beyond time *t* and the observed status at *t*. In the presence of right censored data, the distances must be weighed using an inverse probability of the censoring weight method [34].


Brier score *(t)* = 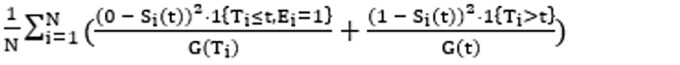



Where *G(t)* = P[censoring time > *t*] (estimated with the Kaplan-Meier estimator on censoring data).

The integrated Brier score (IBS) is simply the average Brier score across all prediction time points.


IBS = 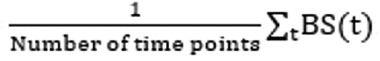



#### Calibration

Calibration of a model refers to the goodness-of-fit of its survival predictions [35]. For example, a model predicts that a patient has a 70% probability of surviving to time t*. Evaluating the model’s calibration aims to answer the question whether the patient can trust this prediction. If 100 patients with identical characteristics as this one were under observation, it would be possible to look at their actual survival times and verify if approximately 70 of them survived to t*. If there was a significant difference between the predicted and observed survival rates, it would mean that the model was not well calibrated [35].

In reality, the data sets are composed of patients with different characteristics. One common method for evaluating a model’s calibration at a chosen time point t* is to stratify all the patients into groups based on the predicted probability of failure by time t*. For example, one method is to stratify the patients into 10 groups, where the cutoff points are the deciles of the distribution of the predicted probabilities. For each group, the observed failure rate by time t* is computed using a Kaplan-Meier estimator fitted to the patients of the group. This observed failure rate is then compared with the average predicted probability of failure by time t* for all patients in the group. The resulting pairs of predicted and observed values can be visually examined side-by-side or on a plot. This process can be repeated for all time points [36].

However, Harrell [37] argued that the binning of the predicted probabilities leads to a loss of precision. To address this issue, Austin et al [36] proposed using regression splines to model the observed failure rate as a function of the complementary log-log transformation of the predicted failure rate, using the relationship: 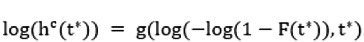. For a visual evaluation of the calibration at a time t*, an estimate of the observed failure probability before t* for every predicted failure probability F_i_(t*) can be obtained using the regression splines, and the resulting pairs can be plotted. With a perfectly calibrated model, this would yield a diagonal curve.

One of the suggested metrics for numerically assessing the calibration is the integrated calibration index (ICI) [38], which is simply the mean absolute difference between the predicted and estimated observed values.


ICI (t*) = 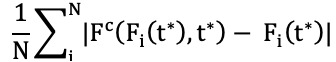



### Development of the Technological Solution

The developed end-user application provides the relevant graft survival probabilities in 3 steps. First, users must enter the required information related to the donor and the transplant candidate (Multimedia Appendix 1). Second, the predictive model is run to obtain survival probabilities under 3 simulated scenarios: the recipient receives the deceased donor kidney (as per the input of step 1), the recipient receives a kidney from a predefined average DCD donor, and the recipient receives a kidney from a predefined average NDD donor. Third, the graft survival predictions are shown (Multimedia Appendix 1). The average DCD and NDD results at the current time point are included to enable the comparison between multiple donor-recipient matches and to support medical decision-making about accepting the proposed donor kidney or waiting for the next available one.

### Software Used for the Project

JIRA (project management; Atlassian), Bitbucket (code management; Atlassian), Confluence (documentation management; Atlassian), Azure (Microsoft) Cloud Platform (cloud), Azure Machine Learning (computations), Google Suite, Teams (team communication), Azure Secured Virtual Machine (data security), VS Code (Microsoft), Python (ML model design and coding), and Expo.io (framework for client web applications, expo.dev) were the software used for the project.

### Code and Model Availability

The code and the trained model can be available upon request if permission from Health Canada and SRTR is obtained in each particular case, which is needed for ethical considerations.

## Results

### Characteristics of the Data Sets

The initial data sets contained information on 210,688 first-time kidney transplant recipients from deceased donors and included 402 variables. The final data set obtained after data cleaning and selection of variables contained data on 180,141 transplants (154,292 from NDD donations and 25,849 from DCD donations) and included 35 variables. Feature selection methods such as LassoCV, ElasticNetCV, and recursive feature elimination did not recommend changing the set of variables chosen based on manually set exclusion criteria. After one-hot encoding of the categorical variables, the total number of input covariates was 170 (Multimedia Appendix 1).

**Figure 2:**
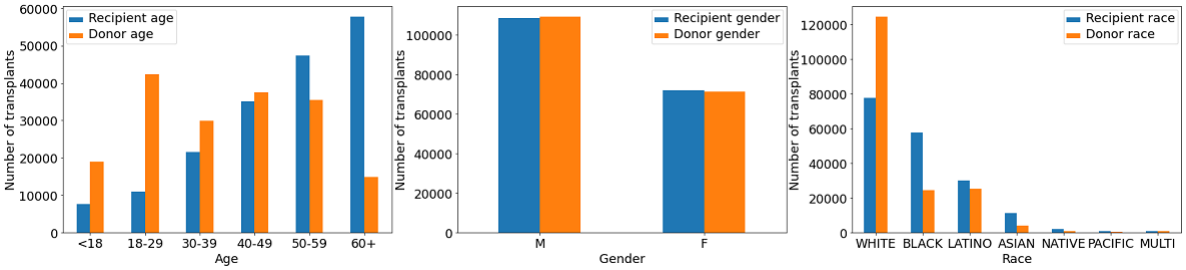
Sociodemographic characteristics of kidney donors and recipients.

Demographics of the patients are shown in Figure 2. This study considered donor-recipient pairs of all ages, including pediatric patients (aged <18 years). The data set contained an unequal number of donors and recipients belonging to different sociodemographic groups. The number of kidney transplant recipients increased with age, which may reflect the fact that the older population is more likely to have end-stage kidney disease. In contrast, the fewest number of eligible donors per age group was the ≥60 cohort. This may also be attributed to the fact that not all kidneys retrieved from the older adult donors are viable. Older adult donors are likely to have more comorbidities, making them illegible to donate. The study population included a large number of male recipients and donors. It was also imbalanced regarding racial groups, with a predominant number of White donors over donors of other races, as well as an unequal number of recipients of different races.

### Choice of Hyperparameters and Training

The 3 neural network–based models were trained using the Adam optimizer with a learning rate of 0.001 and batch size of 128. The optimal number of hidden layers and the number of nodes in the layers were determined separately for each model by testing a range of possible values, starting with small networks and gradually increasing their size. In the 3 cases, increasing the number of hidden layers in the past 3 models resulted in overfitting and decreased discriminative performance. Batch normalization and dropout with a rate of 0.10 were used. In addition, L2 regularization with a factor of 0.001 was used for the RNN model.

DeepSurv consists of 2 dense layers, with 32 and 16 neurons in layers 1 and 2, respectively. DeepHit consists of 3 dense layers with 64, 32, and 16 neurons, respectively. The long short-term memory layers of RNN contain the same number of neurons.

The RSF consists of 100 trees, with a maximum depth of 25 nodes. At each node, 13 randomly selected covariates were considered to split (the square root of the number of covariates). The minimum number of samples required to split a node was 400, and the minimum number of samples in the leaf nodes was 200. Adding more trees did not increase the discriminative ability of the model, and reducing the minimum number of samples to split resulted in overfitting.

### Comparison of RNN Loss Functions

Different loss functions (or objective functions) were tested when building the RNN model. It was found that using the ranking loss proposed in DeepHit [25] yielded a model with better discrimination ability. With the deep recurrent survival analysis [28] loss function, the average C-index was 0.64 on the graft survival task, whereas with the DeepHit ranking loss, the C-index averaged approximately 0.66. Therefore, the latter loss function was used to train the proposed RNN model.

Definition of the loss function:



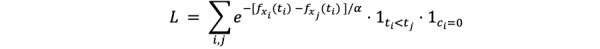



where



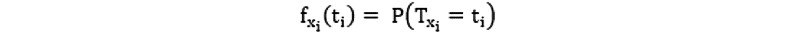

*α*=.1 (a calibration parameter) and

*c_i_*=0 indicates that patient *i* experienced the event of interest during observation period.

Using this loss function to train the neural network yields a model with good discrimination ability but produces poorly calibrated survival predictions. This is because the loss function was mainly designed to encourage the correct ordering of pairs. This issue motivated the postprocessing of the RNN outputs, which is presented in the *Survival Analysis Models* section.

### Model Performance

In preliminary experiments, 3 approaches were tested to obtain survival predictions for DCD kidney transplants with survival analysis models. DeepHit was used as a benchmark for this purpose. The first method was to train the model using only the DCD transplant data, which yielded an average C-index of 0.604. The second method was to train the model using data from both NDD and DCD transplants, which yielded an average C-index of 0.631 on the DCD evaluation set. The third method was to use transfer learning, which consisted of training the model on the larger NDD transplant data set (to gain general knowledge on kidney transplants), then training the model a second time on the DCD transplant data set to gain knowledge specific to DCD grafts. This approach yielded an average C-index of 0.625 on the DCD-only evaluation set. Thus, the model trained only on DCD transplants yielded the poorest results, which may be explained by the lower volume of data available for this specific transplant cohort. The best performance was obtained with the model trained on a data set that included both NDD and DCD transplants. Therefore, further development of ML models was based on the combined data set.

For the final evaluation of the models, a 5-fold cross-validation was used. It consists of randomly splitting the data set into 5 partitions of equal size and repeating the training and evaluation process 5 times, each time using one partition (20%) as the evaluation set and the remaining (80%) as the training set. Table 1 presents the evaluation results for the 5 models that were explored. The C-index obtained by using the Cox proportional hazards model was 0.646. The decision tree–based RSF had a time-dependent C-index of 0.644, whereas the neural network–based models (DeepSurv, DeepHit, and our proposed RNN) obtained time-dependent C-indexes of 0.650, 0.661, and 0.659, respectively. Table 1 also presents IBS and ICI for the 1-year, 5-year, and 15-year time points. The ICI for each time point was the lowest for the Cox proportional hazards model, whereas the C-index and IBS showed the best values for DeepHit and RNN, respectively.

**Figure 3:**
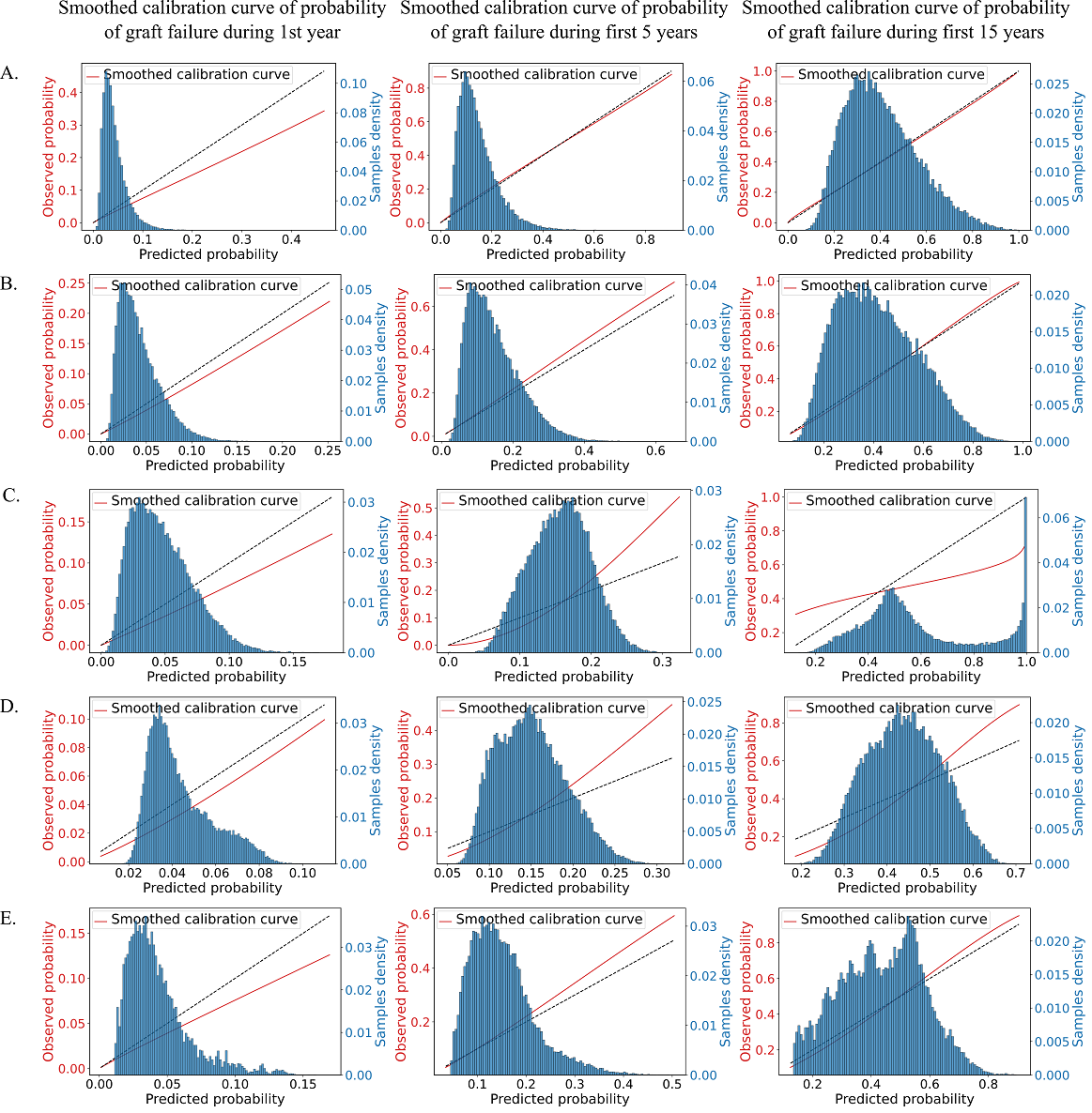
Calibration plots for the probability of graft failure in the first 1, 5, and 15 years following transplant, on the evaluation data.

Figure 3 shows the smoothed calibration curve for the cumulative probability of graft failure at 1 year, 5 years, and 15 years. These plots help to visualize the discrepancy between the graft failure probability predicted by the model and the observed graft failure rate.

For the probability of graft failure in the first year, all 5 tested models had similar calibration, as shown by the ICIs in Table 1 and the calibration curves shown in Figure 3. They all tended to slightly underestimate the survival rate. There were more significant differences in the calibration of the models for the probabilities of graft failure in the first 5 and 15 years. The calibration curves for Cox and DeepSurv are almost perfectly aligned with the identity line and have very low ICIs, indicating that the 2 models produce the most reliable individual survival predictions.

In the case of DeepHit, it is interesting to see that although it had the best discriminative ability, the model failed to produce sufficiently accurate survival predictions, especially at later time points. For the 5- and 15-year time points, DeepHit had the worst ICI (0.0285 and 0.1356) of all models, and its calibration curve had the most significant deviation from the identity line.

The survival predictions produced by the RNN were better calibrated than those produced by DeepHit and RSF. However, as seen on the calibration plots, they are not as well calibrated as those obtained using the Cox and DeepSurv models.

**Table 1:** Evaluation results for the tested machine learning models.

Model	C-index	IBS^a^	ICI^b^ for 1 year	ICI for 5 years	ICI for 15 years
Cox proportional hazards	0.646	0.15439	*0.00942^c^*	*0.00949*	*0.00748*
DeepSurv	0.650	0.15361	0.00957	0.00999	0.01189
DeepHit	*0.661*	0.15259	0.01171	0.02858	0.13561
RSF^d^	0.644	0.15288	0.01058	0.01739	0.04559
RNN^e^	0.659	*0.15220*	0.00989	0.01076	0.02634

^a^IBS: integrated Brier score.

^b^ICI: integrated calibration index.

^c^The italicized values represent the best result obtained for each evaluation metric.

^d^RSF: random survival forest.

^e^RNN: recurrent neural network.

## Discussion

### Overview

This study focused on the development of an ML-based decision support solution to help kidney transplant practitioners and their patients make informed decisions when a deceased donor kidney becomes available. All stages of the development process are described: data acquisition and preparation, evaluation of existing survival analysis models, development and evaluation of a new survival analysis model, and deployment of the technological solution of TRL-4.

### Principal Findings

When building survival analysis models in the context of kidney transplantation, there are several factors that characterize the models and ultimately influence the final quality of the prediction tool.

One factor is the size of the data sets used to build these models. It varies widely between studies, ranging from 80 [39] to 131,709 transplants [16]. It has been demonstrated that large sample sizes improve the predictive performance of ML models [40]. Another important factor is the period for which the risk of mortality or graft failure is predicted. This may depend on data availability and duration of the observation period. Mark et al [22] built an ensemble model to predict patient survival throughout the first 5 years following kidney transplantation. Luck et al [16] evaluated the graft survival probability at each anniversary date of the graft for 15 years following transplantation. Our study was based on the most recent available data and included up to 19 years of observations of 180,141 transplant procedures. The models presented here evaluate graft survival probabilities at each quarterly anniversary of the graft for 15 years. To the best of our knowledge, this is the largest data set with the longest observation period used to build ML models for predictions in the kidney transplantation area.

The performance of a predictive model is also strongly dependent on incorporating prognostically significant variables into the models. The number of variables used for survival analysis in the literature ranges from 6 to several hundred [16,21,41,42]. Selection of a very small number of variables may lead to the exclusion of important factors that may influence the outcome of the transplantation, whereas including a very large number of variables may increase the sparsity of the data, which in turn may cause overfitting. In this study, variables were selected based on medical expertise, previous studies [18,22], and characteristics such as data completeness and data duplication for the first step (35 variables).

The choice of a survival analysis model is also critical. Multiple options have been described in the literature, such as the Cox regression model [18], decision trees [43], support vector machines [44], Bayesian belief networks [12], RSF [22], and artificial neural networks [16,21].

In this study, 5 different models were explored: a regression-based Cox proportional hazards model; RSF; and 3 neural network models, namely, DeepSurv, DeepHit, and a proposed RNN. To the best of our knowledge, the latter was used on kidney transplantation data for the first time in this study. These models were evaluated on the task of predicting kidney graft survival throughout the first 15 years following transplantation. Three metrics were used to evaluate each model: the C-index, IBS, and ICI, along with calibration plots.

### Evaluation of ML Models

The results for the C-index metric shown in Table 1 indicate that the neural network–based models (DeepSurv, DeepHit, and RNN) had better discriminative ability than the Cox model and RSF. In fact, the DeepHit model and our proposed RNN model performed best with a C-index of 0.661 and 0.659, respectively. This indicates their ability to discern groups of donor-recipient pairs that were at a higher risk of experiencing graft failure after transplant from groups that had a lower risk. The improvement compared with the widely used Cox model (C-index of 0.646) may be because of the higher capacity for feature extraction by the neural networks.

The main drawback of the Cox proportional hazards model and DeepSurv is the assumption that the computed hazard ratio is time invariant. In contrast, DeepHit and RNN make no assumptions about the distribution of time-to-event data and can learn the time-varying effects of covariates, making them more flexible. This is important when evaluating survival over a wide time frame, as in our study, over 15 years. For example, a covariate could have a negative effect on survival in the first few years after transplantation but no impact in the later years.

Previously published articles on the prediction of survival of kidney grafts from deceased donors often described different evaluation metrics, such as accuracy [15,44], mean relative absolute error, root mean square error, mean absolute error [15], and C-index [14,16,18], which makes it difficult to perform a comparison between the studies.

The performance of the proposed DeepHit and RNN models evaluated with the C-index is comparable with the previously published iChooseKidney technological solution (0.6640 at 3 years after transplantation) [14] and slightly exceeds the performance of the deep learning survival model described by Luck et al [16] (0.6550). However, the comparison of models based on the C-index alone is limited to the evaluation of their discriminative ability and does not consider the average accuracy of the survival predictions. Making use of ICI and smoothed calibration curves [31,32] helped shed light on the model’s predictive quality.

From the results presented in Table 1 and Figure 3, we can see that there is often an imbalance between a model’s discriminative ability and its calibration. As discriminative ability is required to differentiate between high-risk and low-risk kidney transplants, one might prefer a model with a higher C-index if a comparison of donor-candidate pairs is to be performed, for example, in the case of organ allocation. In contrast, as good calibration is required to provide reliable graft survival predictions, a model with better calibration may be preferable in cases where personalized expected graft survival distributions are to be presented, for example, to a transplant candidate.

### Characteristics of the Developed Technological Solution

We developed a client web application to predict organ survival probability for each potential kidney donor-recipient pair for a period between 1 and 15 years after the transplantation. We opted to use the proposed RNN model to deploy our prototype application. This model offers a compromise between the good discriminative ability and the calibration necessary for the purpose of our application. Indeed, one of the main uses of the decision support application is to simultaneously present graft survival probabilities to a kidney transplant candidate and to offer a point of comparison by presenting graft survival predictions that the patient could expect with other potential donors.

It would be possible to use an alternative approach for computing the predictions at the time *now + average time before a new kidney is available*. To achieve this purpose, it would be necessary to compute the survival for every possible additional wait time and the probability of that wait time occurring, along with the patient survival to that wait time. This could be an objective for future studies.

The presented choice of approach to evaluate the *average donor* predictions at the same time *now* as the predictions for the offered donor kidney is a matter of simplicity and an effective way for patients without statistical background to look at 2 options (accept or refuse the transplant) and understand the possible outcomes.

The client application is at the prototype stage (TRL-4), aiming to demonstrate the capabilities of the ML predictive model. The following information about the candidate recipient is entered in the first step of the application: age, height, weight, ethnicity, sex, diagnosis, number of years on dialysis, presence of diabetes, and presence of angina. The details about potential donor that are entered in the next step are donor’s age, height, weight, ethnicity, donation type, creatinine level, history of diabetes, hypertension diagnosis, hepatitis C diagnosis, and smoking habit. These covariates are used as input for the trained RNN model. In the next step, the user selects the number of years for the prediction target. The output page displays the probability of survival of the transplant for the given donor-recipient pair and specified period as well as for the candidate recipient and average NDD and DCD donors for comparison. It is also possible to expand the result boxes to obtain a detailed view of the results for any specific transplant prediction.

### Future Perspectives

The current application is recipient-oriented and specific to kidney transplantation. Future research could expand this application to other transplanted organs and nonrecipient users. For example, if connected to a candidate database, the application can produce an ordered list of optimal donor-recipient matches when an organ becomes available. The Expo.io development environment for the client was chosen for its capability to support web, Android, and iOS environments, leaving many options open for the distribution and accessibility of the service. The client also connects to the model by using an application programming interface. Thus, although the initial prototype was entirely run in a local environment, the solution could easily be transferred to a cloud-based environment.

In the future, the application could also be extended to include additional predictive models to further inform patients. For example, when a kidney is offered to a patient, it would be instructive to predict the expected waiting time before a *better* kidney becomes available should the patient decide to remain on the waiting list. The solution could also be upgraded to enable the recommendation of the best candidate recipient for each newly available kidney from the existing candidate waiting list based on the predicted graft survival.

### Limitations

Our study has certain limitations, which are important to mention. A built-in selection bias exists in the SRTR data set. It is evident that deceased donor kidneys accepted for transplantation have superior characteristics than those that were never used for transplantation and therefore do not appear in the data. The data were imbalanced according to different age, sex, and racial groups. These selection biases may negatively affect the accuracy of predictions made for candidate recipients or donors who fall into underrepresented populations.

Another limitation is the level of detail available in the data set. The registry-level data from the SRTR certainly does not encapsulate all the characteristics of the clinical and functional status of donor-recipient pairs. Consequently, there must be factors that influence graft survival that were not present in the data. We also did not consider HLA typing, an important variable when matching donors and recipients, because of the complexity of modeling HLA mismatches. We must also consider the population of the United States, on which the models were built. Multiple factors, such as age, race, and state of residency, may reflect the socioeconomic status of patients, which itself may affect access to health care. To use the models built in this study in other countries, for example, in Canada, one must consider that some factors may differently affect graft survival.

### Conclusions

We analyzed and tested 5 ML models to predict kidney graft survival for a period of up to 15 years after transplantation. This study focused on patients who received deceased donor kidney transplants in the United States between 2000 and 2019 and included both NDD and DCD transplants. The resulting RNN predictive model was integrated into a decision support application designed to help kidney transplant practitioners and their patients make informed decisions regarding transplant options.

## Appendix

Multimedia Appendix 1 Variables included in the machine learning models training. The original categories, the CAN_DGN variable (Candidate kidney diagnosis) and REC_FUNCTN_STAT (Candidate functional status), from the SRTR data set were grouped according to the previous work of Mark et al [22].

## Abbreviations

DCD: donation after circulatory death
IBS: integrated Brier score
ICI: integrated calibration index
ML: machine learning
NDD: neurological determination of death
RNN: recurrent neural network
RSF: random survival forest
SRTR: Scientific Registry of Transplant Recipients
TRL-4: technology readiness level 4
