# Supplementary material for: Machine Learning Support for Decision-Making in Kidney Transplantation: Step-by-step Development of a Technological Solution
Source: JMIR Med Inform. 2022 Jun 14;10(6):e34554. doi: 10.2196/34554 (PMC9240927; doi:10.2196/34554)
Supplement: Multimedia Appendix 1 [file medinform_v10i6e34554_app1.docx]

**Multimedia Appendix 1.** Variables included in the machine learning models training. The original categories, the CAN_DGN variable (Candidate kidney diagnosis) and REC_FUNCTN_STAT (Candidate functional status), from the SRTR data set were grouped according to the previous work of Mark et al [22].

| **Variable** | **Type** | **Description** | **Categories** |
| --- | --- | --- | --- |
| DON_AGE | Numeric | Donor age | N.A. |
| DON_HIST_HYPERTEN | Categorical | Donor history of hypertension | No,  Yes, 0-5 years,  Yes, 6-10 years,  Yes, >10 years,  Yes, unknown duratioon |
| DON_HIST_DIAB | Categorical | Donor history of diabetes | No,  Yes, 0-5 years,  Yes, 6-10 years,  Yes, >10 years,  Yes, unknown duratioon |
| DON_BMI_NEW | Numeric | Donor body mass index | N.A. |
| DON_CREAT | Numeric | Donor creatinine level (mg/dL) | N.A. |
| DON_HIGH_CREAT | Categorical | Donor creatinine is > 1.5 mg/dL | Yes, No |
| DON_ANTI_HCV | Categorical | Donor hepatitis C. antibody | Yes, No, Unknown |
| DON_CAD_DON_COD | Categorical | Donor cause of death | Anoxia, Cerebrovascular/stroke, Head trauma, CNS tumor, Unknown |
| DCD_IND_NEW | Categorical | Donation after Circulatory Death | YES, NO |
| ECD_IND | Categorical | Extended criteria donor indicator | YES, NO |
| DON_DEATH_CIRCUM | Categorical | Donor death circumstance | MVA, Suicide, Homicide, Child-abuse, Non-MVA, Death from natural causes, None of the above |
| DON_DEATH_MECH | Categorical | Donor death mechanism | Drowning, Seizure, Drug intoxication, Cardiovascular, Electrical, Gunshot wound, Stab, Blunt injury, SIDS, Intracranial hemorrhages/stroke, Death from natural causes, None of the above |
| DON_GENDER | Categorical | Donor gender | M, F |
| DON_HIST_CIGARETTE | Categorical | Donor cigarette use > 20 Packs years | Yes, No |
| **Variable** | **Type** | **Description** | **Categories** |
| DON_RACE_NEW | Categorical | Donor race | White, Black, Latino, Asian, Native, Pacific, Multi |
| DON_COD_DON_STROKE | Categorical | Donor cause of death is stroke indicator | YES, NO |
| DON_ABO | Categorical | Donor blood type | A, A1, A1B, A2, A2B, AB, B, O |
| REC_AGE_AT_TX | Numeric | Candidate age at time of transplant | N.A. |
| CAN_DIAB_TY | Categorical | Candidate diabetes type | No Diabetes,  Yes, Type I,  Yes, Type II,  Yes, Type Other,  Yes, Type Unknown |
| CAN_BMI_NEW | Numeric | Candidate body mass index | N.A. |
| CAN_ANGINA_NEW | Categorical | Candidate angina indicator | Yes, No |
| CAN_DIALYSIS_YEARS | Numeric | Candidate number of years on dialysis | N.A. |
| CAN_WAITING_TIME_NEW | Numeric | Candidate number of years on waiting list | N.A. |
| CAN_DGN | Categorical | Candidate kidney diagnosis* | Group_1, Group_2, Group_3, Group_4, Group_5, Group_6, Group_7, Group_8 |
| CAN_PREV_TX_NEW | Categorical | Recipient had previous organ transplant indicator | Yes, No |
| CAN_GENDER | Categorical | Candidate gender | M, F |
| CAN_RACE_NEW | Categorical | Candidate race | White, Black, Latino, Asian, Native, Pacific, Multi |
| CAN_STATE_NEW | Categorical | Candidate residence state in the United-States | All 50 states |
| CAN_ABO | Categorical | Candidate blood type | A, A1, A1B, A2, A2B, AB, B, O |
| CAN_DRUG_TREAT_COPD | Categorical | Candidate is treated for chronic obstructive pulmonary disease | Yes, No, Unknown |
| REC_MED_COND | Categorical | Candidate medical condition at time of transplant | In intensive care unit,  hospitalized not in ICU,  not hospitalized |
| **Variable** | **Type** | **Description** | **Categories** |
| REC_FUNCTN_STAT | Categorical | Candidate functional status (level of normal daily activity)* | - Performs activities of daily living with total assistance, - 10-20% Very sick, hospitalization necessary, - 30-50% Requires considerable assistance, but death no imminent, - 60-70% Perfors activities of daily living with some assistance, - 80-100% Performs activities of daily living with no assistance, - N.A. (Patient <1 year old, - Unknown |
| REC_A_MM_EQUIV_TX | Numeric | Number of HLA A mismatches | N.A. |
| REC_B_MM_EQUIV_TX | Numeric | Number of HLA B mismatches | N.A. |
| REC_DR_MM_EQUIV_TX | Numeric | Number of HLA DR mismatches | N.A. |
